# Extremophilic 50S Ribosomal RNA-Binding Protein L35Ae as a Basis for Engineering of an Alternative Protein Scaffold

**DOI:** 10.1371/journal.pone.0134906

**Published:** 2015-08-06

**Authors:** Anna V. Lomonosova, Elena V. Ovchinnikova, Alexei S. Kazakov, Alexander I. Denesyuk, Alexander D. Sofin, Roman V. Mikhailov, Andrei B. Ulitin, Tajib A. Mirzabekov, Eugene A. Permyakov, Sergei E. Permyakov

**Affiliations:** 1 Institute for Biological Instrumentation of the Russian Academy of Sciences, Institutskaya str., 7, Pushchino, Moscow region, 142290, Russia; 2 Department of Biosciences, Åbo Akademi University, Turku, 20520, Finland; 3 Antherix, Institutskaya str., 7, Pushchino, Moscow region, 142290, Russia; 4 Biomirex Inc., 304 Pleasant Street, Watertown, Massachusetts, 02472, United States of America; Centro Nacional de Biotecnologia—CSIC, SPAIN

## Abstract

Due to their remarkably high structural stability, proteins from extremophiles are particularly useful in numerous biological applications. Their utility as alternative protein scaffolds could be especially valuable in small antibody mimetic engineering. These artificial binding proteins occupy a specific niche between antibodies and low molecular weight substances, paving the way for development of innovative approaches in therapeutics, diagnostics, and reagent use. Here, the 50S ribosomal RNA-binding protein L35Ae from the extremophilic archaea *Pyrococcus horikoshii* has been probed for its potential to serve as a backbone in alternative scaffold engineering. The recombinant wild type L35Ae has a native-like secondary structure, extreme thermal stability (mid-transition temperature of 90°C) and a moderate resistance to the denaturation by guanidine hydrochloride (half-transition at 2.6 M). Chemical crosslinking and dynamic light scattering data revealed that the wild type L35Ae protein has a propensity for multimerization and aggregation correlating with its non-specific binding to a model cell surface of HEK293 cells, as evidenced by flow cytometry. To suppress these negative features, a 10-amino acid mutant (called L35Ae 10X) was designed, which lacks the interaction with HEK293 cells, is less susceptible to aggregation, and maintains native-like secondary structure and thermal stability. However, L35Ae 10X also shows lowered resistance to guanidine hydrochloride (half-transition at 2.0M) and is more prone to oligomerization. This investigation of an extremophile protein’s scaffolding potential demonstrates that lowered resistance to charged chemical denaturants and increased propensity to multimerization may limit the utility of extremophile proteins as alternative scaffolds.

## Introduction

Protein engineering for selective target recognition has numerous applications in research, diagnostics and therapeutics [1–12]. Although animal-sourced and bioengineered antibodies have been successfully used for these purposes for decades [6–9], the application of antibodies is often complicated by their relatively large molecular sizes, complex multi-subunit structure, limited stability, and abundance of post-translational modifications requiring the use of eukaryotic expression systems, which together lead to technical challenges and high production costs. To overcome these limitations, alternative/artificial binding proteins (ABPs) have been developed [1–6, 10–12]. ABPs mimic conventional antibodies, but are based on relatively smaller immunoglobulin-like or non-immunoglobulin folds (‘alternative scaffolds’ or ‘alternative protein scaffolds’, APSs). The alternative use of the term ‘protein scaffold’ to refer to proteins involved in assembling signaling proteins into complexes (reviewed in ref. [13]) is not intended in this article.

An engineered alternative protein scaffold usually possesses a compact stable protein frame and polypeptide region(s), which have been subjected to amino acid randomization to provide a broad repertoire (10^5^−10^13^) of polypeptides with structural stability close to that of the original protein. The structures possessing highest affinity to a target of choice can then be selected from such synthetic combinatorial libraries using *in vitro* display technologies. The resulting ABP molecules possess antibody-like specificity and selectivity of interaction with the target. Additional advantages of ABPs over the conventional antibodies include an order of magnitude lower molecular weight and respectively lower molecular sizes, simple subunit structure, minimal post-translational modifications, high stability, applicability of simple and efficient bacterial expression systems, high protein yields, and, respectively, lower production costs. The small size of ABPs ensures an increased tissue and tumor penetration, as well as improved access to grooves on target surfaces normally inaccessible to antibodies [11]. The smaller size of ABPs is also advantageous for selective blocking of specific ligand-binding sites of multi-ligand receptors. Although the low molecular weights of ABPs greatly limit their serum half-life (desired, for example, for tumor imaging applications, but unfavorable for a prolonged therapy), half-life can be extended by fusing ABP molecules with high molecular weight entities [6, 10]. A similar approach can be employed to resolve the issue of absent natural effector functions of antibodies due to the lack of Fc domain [11]. Furthermore, the fusion of ABP molecules or various ABPs derived from the same alternative scaffold results in multivalent or multispecific constructs, respectively [6, 10]. Overall, artificial binding proteins occupy a specific niche in between antibodies and low molecular weight drugs/substances, which paves the way for development of innovative approaches for therapy, diagnostics, and reagents use.

Several dozens of alternative scaffolds, based on either artificial (exemplified by Top7 [14]) or natural proteins, have emerged over the last two decades [1–6, 10–12]. Among the most established of them are: Adnectins (based on the 10^th^ human fibronectin type III domain) [15, 16], Affibodies (based on Fc-binding Z domain derived from staphylococcal protein A) [17, 18], Anticalins (based on lipocalins) [19, 20] and DARPins (based on the ankyrin fold) [21, 22]. Many artificial binding proteins are currently under clinical trials, including Angiocept (CT-322 or BMS-844203; Adnectin inhibiting vascular endothelial growth factor (VEGF) receptor 2 for treatment of advanced solid tumors [23]), ABY-025 (Affibody against human epidermal growth factor receptor type 2 for imaging of this receptor in breast cancer metastases [24]), Angiocal (PRS-050; Anticalin against VEGF-A for treatment of advanced solid tumors [25]), MP0112 (DARPin targeting VEGF for treatment of diabetic macular edema [26] and exudative age-related macular degeneration [27]), further reviewed in [1, 10, 28]. Notably, KALBITOR (Ecallantide or DX-88; Kunitz domain-derived inhibitor of human plasma kallikrein for the treatment of acute hereditary angioedema) has made its way to the market [29–31]. The promising results of alternative scaffold technology indicate that artificial binding proteins may complement or compete with therapeutic antibodies in the rapidly growing multi-billion dollar market. For this reason, further development of validated APSs as well as engineering of novel APSs with superior physico-chemical properties and better prospects in therapeutic and diagnostic applications can be expected.

Since genetic randomization of ‘paratopic’ regions of an alternative scaffold may negatively affect structural stability, APS structure should tolerate multiple amino acid substitutions and even deletions and insertions in these regions [32, 33]. In this respect, proteins originating from extremophilic organisms are of special interest, as their structural stability typically exceeds the respective stability of orthologs from non-extremophilic organisms [34–36]. Some extremophilic proteins have indeed been used as a framework for the construction of artificial binding proteins. For instance, the Sso7d protein from the thermoacidophilic archaea *Sulfolobus solfataricus* [37, 38], Sac7d from the thermoacidophilic archaea *Sulfolobus acidocaldarius* [39, 40], and their relatives with OB-fold domain were patented for use as a basis for engineering of specific binders (WO 2011077395 A1, WO 2008068637 A2). Hence, a broader search for similar proteins from extremophiles represents a promising approach for APS development.

Here we describe a 50S ribosomal protein L35Ae from hyperthermophilic archaea *Pyrococcus horikoshii* (strain ATCC 700860 / DSM 12428 / JCM 9974 / NBRC 100139 / OT-3) (UniProt entry O74099 [41]) that has been explored for its potential to serve as a frame for development of an original alternative scaffold. The L35A/L35Ae is found in archaea and eukaryotes, but not in bacteria. To avoid confusion with various naming of this protein in archaea and some eukaryotes (‘L33’ in yeast) and with bacterial and eukaryotic L35 proteins, the recently proposed nomenclature of ribosomal proteins designates it as ‘eL33’ [42]. Here, we will use the more conventional name, ‘L35Ae’. L35Ae is located within the large ribosomal subunit and binds both initiator and elongator tRNAs [43]. shRNA inhibition demonstrated that L35Ae is essential for maturation of 28S and 5.8S rRNAs, large subunit biogenesis, cell proliferation and survival, and is involved in the pathogenesis of Diamond-Blackfan anemia [44]. L35Ae was shown to confer resistance to chemotherapy, UV-radiation, anti-Fas antibody and serum starvation to Jurkat cells [45]. Since L35Ae is overexpressed in glioblastoma multiforme [46] and downregulated in colorectal carcinoma [47], L35Ae may also play a role in modulating chemoresistance in cancerous cells.

**Fig 1:**
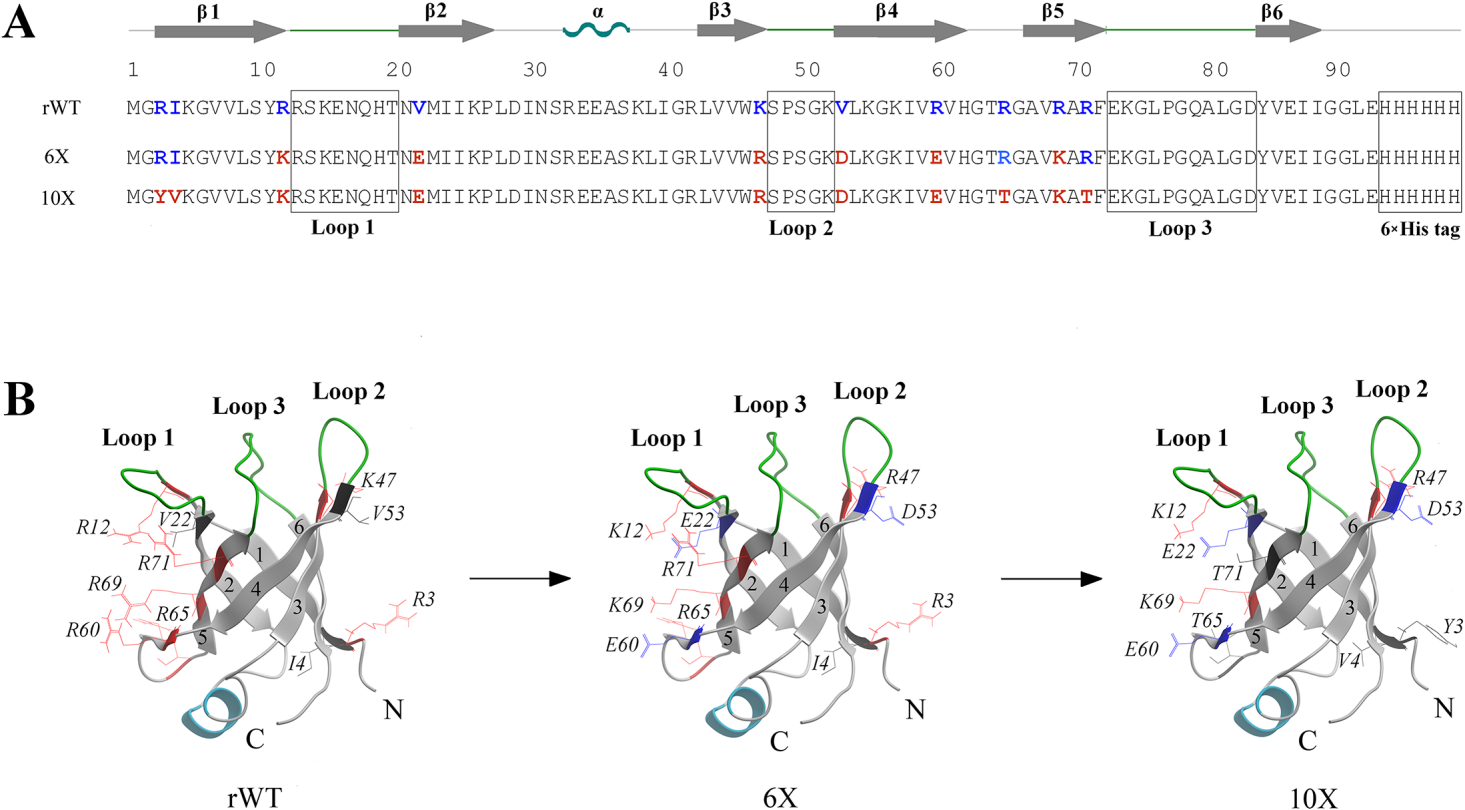
Amino acid sequences (A) and structural models (B) of the recombinant forms of L35Ae from *P*. *horikoshii* used in the present study. **(A)** Secondary structure elements for tertiary structure of L35Ae from *P*. *furiosus* (PDB entry 2lp6 [50]) are indicated. The loop regions mimicking CDRs of immunoglobulins and a 6xHis tag are shown in rectangles. The residues affected by mutagenesis are indicated using bold color font. **(B)** The β-sheets from 1 to 6 (grey) and CDR-like loop regions (green) are indicated. The residues affected by mutagenesis are shown using wire representation. The structural models of L35Ae forms were built based on PDB entry 2lp6 [50]. The figure was created using ICM Browser v.3.7-3b (MolSoft L.L.C.) software.

The L35Ae protein from *P*. *horikoshii* is a small (87 residues, M_r_~10 kDa), basic (pI~11), globular protein that lacks Cys residues [41], which favors its production in bacteria. The tertiary structure of its ortholog from *Pyrococcus furiosus* (UniProt entry Q8TZV6; sequence identity is 93%) reveals a ‘reductase/isomerase/elongation factor common domain’ fold (SCOP entry 50412): a six-stranded anti-parallel β-barrel with a short α-helix located between strands β2 and β3 (Fig 1). Notably, the loop regions between strands β1 and β2, β3 and β4, β5 and β6 (referred to as loops 1, 2 and 3, respectively; Fig 1) closely resemble complementarity determining regions (CDRs) of immunoglobulins. These loops are attractive for diversification as exemplified by APSs based on β-barrel (Anticalin [19, 20]) and β-sandwich folds (Adnectins [15, 16], among others [4]). The suitability of the L35Ae protein from *P*. *horikoshii* for development of an alternative scaffold has been estimated here by means of exploration of the L35Ae features crucial for its use as an APS such as structural stability [32], propensity to multimerization and aggregation [48], and binding to biologically relevant surfaces such as the surface of cultured human cells. The latter feature is vital for selective recognition of therapeutic/diagnostic targets, but typically neglected during APS design. Though recombinant wild type (rWT) L35Ae exhibits a marked tendency to both aggregation and association with mammalian cells, these unfavorable features of the protein have been suppressed by directed mutagenesis of its surface residues. The resulting L35Ae mutant can be used as a framework for further development of an original APS.

## Materials and Methods

### Materials

Gene of 6×His-tagged recombinant wild type 50S ribosomal protein L35Ae from *Pyrococcus horikoshii* (strain *ATCC 700860 / DSM 12428 / JCM 9974 / NBRC 100139 / OT-3*; UniProt entry O74099) was codon optimized for the expression in *E*. *coli* using Optimizer server (http://genomes.urv.es/OPTIMIZER/ [51]) and synthesized by Bio Basic Canada Inc. (Markham, Ontario, Canada):

ATGGGTCGCATTAAAGGCGTGGTGCTGAGCTATCGCCGCAGCAAGGAAAACCAGCATACCAACGTGATGATTATCAAGCCGCTGGACATTAACAGCCGCGAGGAAGCGAGCAAACTGATTGGCCGCCTGGTGGTCTGGAAAAGCCCGAGCGGCAAGGTGCTGAAAGGCAAAATCGTGCGCGTGCATGGCACCCGCGGCGCGGTGCGCGCGCGCTTCGAGAAAGGCCTGCCGGGTCAGGCGCTGGGCGATTACGTGGAAATCATCGGCGGTCTCGAGCACCACCACCACCACCACTGA

The L35Ae gene was cloned into pET-28b(+) vector (Novagen) between the *Nco*I and *Xho*I restriction sites. The final protein sequence, referred to as ‘rWT L35Ae’, contained a Gly residue inserted between the residues M1 and R2 and the C-terminal GGLE sequence, followed by a 6×His tag (see Fig 1A). The amino acid substitutions were introduced into the plasmid using the primers shown in Table 1. Nucleotide sequences of the resulting genes were verified by automatic DNA sequencing.

**Table 1:** List of the primers used for mutagenesis of rWT L35Ae.

Substitution/Primer	Nucleotide sequence
**R12K**	GTTTTCCTTGCTGCGCTTATAGCTCAGCACCACGCCTT
**V22E**	CAGCATACCAACGAGATGATTATCAAGCCGCTGG
**K47R**	GCTCGGGCTTCTCCAGACCACCAGGCGGCCAA
**V53D**	GGCAAGGACCTGAAAGGCAAAATCGTG
**R60E**	GCCGCGGGTGCCATGCACTTCCACGATTTTGCCTTTCAG
**R69K**	GCGGTGAAAGCGCGCTTCGAGAAAGGC
**R3YI4V**	GTTGTTCCATGGGTTACGTTAAAGGCGTGGTGCTGAGC
**R65, 71Tf**	GGAAGTGCATGGCACCACTGGCGCGGTGAAAGCGACCTTCGAGAAAGGCCTGCC
**R65Tb**	CCAGTGGTGCCATGCACTTCC
**T7-terminator**	GCTAGTTATTGCTCAGCGG

*E*. *cloni* 10G electrocompetent cells (*E*. *coli* strain optimized by Lucigen for high efficiency transformation: F^-^ mcr*A* Δ(*mrr-hsd*RMS-*mcr*BC) *end*A1 *rec*A1 ϕ80*d*lacZΔM15 Δ*lac*X74 *ara*D139 Δ(*ara*,*leu*)7697 *gal*U *gal*K *rps*L *nup*G λ- *ton*A) were used for cloning of L35Ae 6X (rWT L35Ae with amino acid substitutions R12K, V22E, K47R, V53D, R60E, R69K) and L35Ae 10X (rWT L35Ae with substitutions R3Y, I4V, R12K, V22E, K47R, V53D, R60E, R65T, R69K, R71T) mutants.

T7 Express *lysY* (High Efficiency) *E*. *coli* strain (MiniF *lysY* (Cam^R^) */ fhuA2 lacZ*::*T7 gene1 [lon] ompT gal sulA11 R(mcr-73*::*miniTn10—*Tet^S^ *)2 [dcm] R(zgb-210*::*Tn10—*Tet^S^ *) endA1 ∆(mcrC-mrr)114*::*IS10*) (New England BioLabs Inc.) was used for expression of rWT L35Ae, L35Ae 6X and L35Ae 10X.

Human Embryonic Kidney 293 (HEK293) cell line was obtained from the Russian Cell Culture Collection (Institute of Cytology of the Russian Academy of Sciences, St. Petersburg, Russia).

KH_2_PO_4_ and Na_2_HPO_4_ were from Panreac Química S.L.U. Sodium acetate, Tris, EDTA, and imidazole were from Sigma-Aldrich Co. Glycine and Tween 20 were purchased from Bio-Rad Laboratories, Inc. Boric acid and guanidine hydrochloride (GuHCl) were from Merck KGaA. KCl was from Reachem (Moscow, Russia). NaCl, components of 2YT media, and molecular mass markers for SDS-PAGE were from Helicon (Moscow, Russia). MgCl_2_×6H_2_O and PMSF were purchased from Amresco LLC. β-D-1-thiogalactopyranoside (IPTG) was from Serva Electrophoresis GmbH. FACS buffer was from Beckman Coulter, Inc. DNAse I and RNAse A were from F. Hoffmann-La Roche Ltd. All restriction enzymes, T4 DNA ligase, T4 polynucleotide kinase, β-agarase I, DNA polymerases, mouse 6x-His Epitope Tag monoclonal antibodies and fetal bovine serum were from Thermo Fisher Scientific Inc. Anti-hCCR7 Fab17 specific to human C-C chemokine receptor type 7 (hCCR7) was from Meprotek (Pushchino, Russia). Anti-mouse IgG–R-Phycoerythrin were from Sigma-Aldrich Co. Glutaraldehyde and SP Sepharose FF were from GE Healthcare. Profinity IMAC (immobilized metal affinity chromatography) Ni-Charged Resin was provided by Bio-Rad Laboratories, Inc. All buffers and other solutions were prepared using either distilled or ultrapure water. SnakeSkin dialysis tubing (3.5 kDa MWCO) was from Thermo Fisher Scientific Inc.

### Methods

#### Calculation of energies of charge-charge interactions

Electrostatics calculations were performed using finite difference Poisson-Boltzmann method as implemented in DelPhi module of the Discovery Studio Software (Accelrys Software Inc.) as described in ref. [52]. The model structures of L35Ae were constructed from the NMR structure of 50S ribosomal protein L35Ae from *P*. *furiosus* (PDB entry 2lp6 [50], Fig 1) using MODELER [53] module of the Discovery Studio. Ionic strength of the solvent (dielectric constant 80.0, molecule radius 1.4 Å) was 0.3 M, pH 8.0. The solute extent was 80%, and number of grid points was 33. Debye-Hückel-type boundary conditions were used. DelPhi’s default charge and radius templates were used. The charges of atoms subjected to estimation of electrostatic potential were selectively set to zero. The total energy of charge-charge interactions was estimated via summing up the electrostatic energies of all charges and division by a factor of 2.

#### Mutagenesis of rWT L35Ae

The L35Ae 6X mutant (Fig 1) was prepared by PCR using pET-28b(+) vector with rWT L35Ae gene as a template. The PCR reaction mixture (50 μl) contained 25 ng of the template, 5 μl of 10× PfuUltra reaction buffer, 2.5 units of PfuUltra DNA polymerase, 0.2 mM deoxynucleotide triphosphates (dNTPs), 10 pmol of R60E and R69K primers (Table 1). The PCR conditions: 94°C for 4 min; 25 cycles of 25 s at 94°C, 10 s at 55°C, and 2 min at 72°C; 5 min at 72°C. The PCR product was treated with 10 units of *Dpn* I for 2 h at 37°C, followed by gel purification and treatment with β-agarase I. The mixture of 10 μl of the resulting solution, 1.2 μl of 10x T4 DNA ligase buffer, 2 Weiss units of T4 DNA ligase, 3 units of T4 polynucleotide kinase was incubated for 2 h at room temperature. The mixture was transformed into *E*. *cloni* 10G electrocompetent cells, plated onto 2YT agar supplemented with 50 μg/ml kanamycin and 1% glucose for further plasmid extraction. The sequence-verified plasmid was used as a template for introduction of K47R+V53D and R12K+V22E mutations following the described procedure. The resulting plasmid with L35Ae 6X gene was used for cloning into T7 Express *lysY* (High Efficiency) *E*. *coli* strain or for further L35Ae mutagenesis.

The L35Ae 10X mutant (Fig 1) was prepared by PCR using the pET-28b(+) vector with L35Ae 6X gene as a template. Two PCR reactions were performed using the following pairs of primers: R3YI4V –R65Tb or (R65, 71Tf)–(T7-terminator) (Fig 1). 25 μl of the reaction mixture contained 25 ng of the template, 2.5 μl of 10× Taq DNA polymerase PCR buffer, 1 unit of Taq DNA polymerase, 0.2 mM dNTPs, 5 pmol of each of the primers. The PCR conditions: 94°C for 4 min; 20 cycles of 25 s at 94°C, 25 s at 55°C, and 40 s at 72°C; 5 min at 72°C. The two PCR products were treated with 10 units of *Dpn* I at 37°C for 2 h, gel-purified and treated with β-agarase I. Next PCR reaction mixture (50 μl) contained equimolar mixture of the two PCR products (20 μl each), 5 μl of 10× Taq DNA polymerase PCR buffer, 2 units of Taq DNA polymerase, 0.2 mM dNTPs, 10 pmol of each of R3YI4V and T7-terminator primers. The same PCR conditions were used. The resulting PCR product was gel-purified, treated with β-agarase I and digested with *Nco*I and *Xho*I enzymes. It was then cloned into pET-28b(+) vector predigested by *Nco*I and *Xho*I. The ligated plasmid was transformed into *E*. *cloni* 10G electrocompetent cells, plated onto 2YT agar supplemented with 50 μg/ml kanamycin and 1% glucose for further plasmid extraction.

#### Expression and purification of recombinant L35Ae

Electrocompetent T7 Express *lysY* (High Efficiency) *E*. *coli* cells were transformed with L35Ae plasmid and plated on 2YT agar plate supplemented with 50 μg/ml kanamycin and 1% glucose. A colony grown at 37°C for 18 h was inoculated into 8 ml of 2YT medium with 50 μg/ml kanamycin and 1% glucose, and grown for 16 h at 37°C, shaking at 250 rpm. The resulting culture was inoculated into 800 ml of 2YT medium with 50 μg/ml kanamycin and grown at 37°C, shaking at 250 rpm, until the optical density at 600 nm reached 0.5 AU. L35Ae expression was induced by 0.5 mM IPTG. The cells were grown for 3 h, harvested by centrifugation at 3,800 × g for 20 min at 4°C, resuspended in 80 ml of lysis buffer (PBS, 150 mM NaCl, 0.5 mM PMSF, 1 mM EDTA, 5 mM β-mercaptoethanol, 0.1% Tween 20, pH 7.0) and disintegrated using a French press (IBI RAS, Pushchino, Russia). The lysate was incubated with 80 units of RNase A at 37°C for 15 min, treated with 80 units of DNase I (20 mM MgCl_2_) at 37°C for 15 min and centrifuged at 8,200 × g for 25 min at 4°C. The 6×His-tagged L35Ae was extracted from the cleared supernatant using 5 ml of Profinity IMAC Ni-Charged Resin: the supernatant was incubated with the medium for 2 h under gentle shaking at room temperature. The medium was packed into the column and the bound L35Ae was eluted with PBS, 150 mM NaCl, 30 mM EDTA, pH 7.0 buffer, and dialyzed at 4°C against the 100-fold volume of 10 mM sodium acetate, 300 mM NaCl, pH 5.3 buffer (buffer A). The dialyzed solution was cleared by centrifugation at 15,500 × g for 20 min at 4°C and loaded at a flow rate of 1 ml/min onto SP Sepharose FF column (3 ml) equilibrated with buffer A. The column was washed with 30 ml of buffer A. The protein was eluted with 30 ml of 10 mM sodium acetate, 1 M NaCl, pH 5.3 (flow rate of 1 ml/min). The homogeneity of L35Ae sample was confirmed by 12% Tris-glycine SDS-PAGE and staining with Coomassie Brilliant Blue R-250. The purified protein was exhaustively dialyzed at 4°C against distilled water, freeze-dried and stored at -18°C. L35Ae samples were analyzed after dissolution of the freeze-dried protein in an appropriate buffer as soon as possible (typically, within 0.5–2 h). The wild type and mutant L35Ae samples were treated in an identical manner to ensure highest quality of the comparative study. Protein concentrations were measured spectrophotometrically using molar extinction coefficient at 280 nm calculated according to ref. [54]: 8,480 M^-1^cm^-1^ for rWT L35Ae and L35Ae 6X, and 9,970 M^-1^cm^-1^ for L35Ae 10X. The yield was 40 mg and 60 mg of protein per liter of cell culture, for rWT L35Ae and (L35Ae 6X)/(L35Ae 10X) samples, respectively.

#### Circular dichroism

Far-UV circular dichroism (CD) studies were carried out at 25°C using a J-810 spectropolarimeter (JASCO) equipped with a Peltier-controlled cell holder. The instrument was calibrated with an aqueous solution of d-10-camphorsulfonic acid (JASCO) according to the manufacturer’s instruction. The cell compartment was purged with nitrogen. The quartz cells with pathlength of 1.0 mm were used; protein concentration was 11–12 μM. The contribution of buffer (5 mM Na_2_HPO_4_, 0.88 mM KH_2_PO_4_, 150 mM NaCl, 1.35 mM KCl, pH 7.0) was subtracted from experimental spectra. Spectral bandwidth was 1 nm, averaging time 2 s, and accumulation 3. The contents of α-helices and β-sheets were estimated using CDPro software package [55] (http://lamar.colostate.edu/~sreeram/CDPro/main.html).

#### Scanning calorimetry

Differential scanning calorimetry (DSC) studies were carried using a Nano DSC microcalorimeter (TA Instruments) at a 1 K/min heating rate and an excess pressure of 4 bars (20 mM H_3_BO_3_–NaOH, 300 mM NaCl, pH 8.5). rWT L35Ae and L35Ae 10X concentrations were 0.65 mg/ml and 1.5 mg/ml, respectively. The protein specific heat capacity was calculated as described by Privalov and Potekhin [56]. The partial molar volume of L35Ae samples was estimated according to ref. [57]. The temperature dependence of excess specific heat capacity of the protein (*ΔC*_*p*_) was calculated as described in ref. [58]. The calorimetric enthalpy of protein denaturation, *ΔH*_*cal*_, was calculated by integration of temperature dependence of *ΔC*_*p*_. The mid-transition temperature, *T*_*o*_, was estimated from fitting of temperature dependence of *ΔC*_*p*_ by a single Gaussian function using OriginPro 8.0 software (OriginLab Corporation, Northampton, MA, USA).

#### GuHCl-induced unfolding of recombinant L35Ae

GuHCl-induced unfolding of L35Ae samples was studied by monitoring GuHCl-dependent changes in position of intrinsic fluorescence spectrum maximum (λ_max_). Measurements were performed on a Cary Eclipse spectrofluorimeter (Varian Inc.), equipped with a Peltier-controlled cell holder. Quartz cells with pathlength of 10 mm were used. Protein concentration was 3 **μ**M. Buffer conditions: PBS, 150 mM NaCl, pH 7.0. The L35Ae samples were incubated with GuHCl for 2 h at room temperature prior to the measurements. The fluorescence of a single tryptophan of L35Ae was excited at 280 nm. Spectral bandwidths were 2.5 nm and 10 nm for excitation and emission monochromators, respectively. All spectra were corrected for spectral contribution of the buffer, spectral sensitivity of the instrument and fitted to log-normal curves using LogNormal software (IBI RAS, Pushchino, Russia) according to ref. [59] using nonlinear regression analysis [60]. λ_max_ values were obtained from these fits. The GuHCl-dependent changes in fluorescence intensity at 314 nm were fitted by Boltzmann function using OriginPro 8.0 software (OriginLab Corporation, Northampton, MA, USA):

$$F_{314nm}=\frac{F_{init}-F_{final}}{1+e^{([GuHCl]-{\left[GuHCl\right]}_{1/2})/\Delta }}+F_{final}$$ (1)

Here, *[GuHCl]*_*1/2*_ is the mid-transition GuHCl concentration, *Δ* is a factor reflecting width of the transition, while *F*_*init*_ and *F*_*final*_ are the fluorescence intensities corresponding to GuHCl-free and GuHCl-saturated protein forms, respectively.

#### Dynamic light scattering studies

Dynamic light scattering (DLS) measurements were performed at 25.0°C using a Zetasizer Nano ZS instrument (Malvern Instruments Ltd.). The protein solutions (1.5 mg/ml in mM PBS, 150 mM NaCl, pH 7.0) were filtered using 10 nm Whatman Anotop 10 syringe filters. The backscattered light from a 4 mW He-Ne laser (632.8 nm) was collected at an angle of 173°. The acquisition time for a single autocorrelation function was 100 s, the resulting autocorrelation functions were averaged values from 10 measurements. The intensity distributions of particle sizes were calculated using the following parameters: a refractive index of 1.330 and a viscosity value of 0.8882 cP. The apparent molecular weights of recombinant L35Ae samples were calculated using Mark-Houwink equation, as implemented for proteins in Zetasizer Nano ZS software.

#### Chemical crosslinking of recombinant L35Ae

Crosslinking of L35Ae samples (0.3–1 mg/ml) with 0.05% glutaraldehyde was performed at 37°C for 1 h (PBS, 150 mM NaCl, pH 7.0). The reaction was quenched by addition of the SDS-PAGE sample loading buffer. 5 μg of the sample were analyzed by SDS-PAGE (4–15%). The gels were stained with Coomassie Brilliant Blue R-250, scanned using Molecular Imager PharosFX Plus System (Bio-Rad Laboratories, Inc.) and analyzed by Quantity One software.

#### Gel-filtration chromatography of L35Ae samples

10 μl of rWT L35Ae (3.5 mg/ml) or L35Ae 10X (3.7 mg/ml) solutions in PBS, 150 mM NaCl, pH 7.0 buffer were applied to Superdex 75 10/300 GL column (GE Healthcare). The protein elution was performed using D-7000 HPLC system (Hitachi) at flow rate of 0.4 ml/min and room temperature. The elution process was monitored by absorbance at 280 nm. The column was calibrated using bovine albumin (65 kDa and 130 kDa), chicken albumin (44 kDa), bovine β-lactoglobulin (36 kDa), bovine carbonic anhydrase (29 kDa), bovine α-lactalbumin (14.2 kDa) and bovine aprotinin (6.5 kDa) as molecular mass standards. The dependence of protein elution time on logarithm of its molecular mass was plotted for the mass standards and linearly fitted. The apparent molecular weight of L35Ae sample was determined by comparison of its elution time with the calibration line.

#### Analysis of L35Ae interaction with HEK293 cells

The stock solution of L35Ae (1 μM) in FACS buffer (PBS, 150 mM NaCl, pH 7.0, 2% calf serum, 0.01% NaN_3_) was sequentially diluted with FACS buffer. The HEK293 cells prewashed with FACS buffer were incubated on ice with different concentrations of L35Ae (overall, six L35Ae concentrations in the range from 1 μM to 30 pM) for 1 h. Then the cells were washed by FACS buffer and incubated with mouse 6x-His Epitope Tag monoclonal antibody (Thermo Fisher Scientific Inc.) for 1 h. The cells were again washed with FACS buffer and then incubated with anti-mouse IgG–R-Phycoerythrin for 1 h. Then the cells were rinsed with FACS buffer and analyzed using Guava PCA-96 flow cytometer (Guava Technologies Inc., USA). Fluorescence excitation and emission were at 532 nm and 580–583 nm, respectively; U = 425 V, FSC gain of 8, medium flow rate. 6×His-tagged anti-hCCR7 Fab17 (Meprotek, Pushchino, Russia) was used as a negative control.

## Results

### Structural properties of rWT L35Ae

The rWT L35Ae protein (UniProt entry O74099 with a Gly residue inserted between the residues M1 and R2 and the C-terminal GGLE sequence, followed by a 6×His tag–see Fig 1A), was expressed in *E*. *coli*, isolated and purified to homogeneity, and characterized with respect to its secondary structure, stability of tertiary structure, and propensity to multimerization and aggregation.

The far-UV circular dichroism (CD) analysis revealed that rWT L35Ae was mostly β-pleated with a β-sheet content of 40% (Table 2). Its close homologue (sequence identity of 93%), L35Ae from *P*. *furiosus*, exhibits similar secondary structure with a corresponding predominance of β-sheets (β-sheet content of 45%, as calculated from PDB entry 2lp6 [50] using DSSP algorithm as implemented in PDB).

**Table 2:** Structural properties of L35Ae samples.

L35Ae sample	CD		DSC		GuHCl	DLS	
	α-helices, %	β-sheets, %	T_0_, °C	ΔH_cal_, J/g	[GuHCl]_1/2_, M	R_h_, nm	MW/MW_m_
**rWT**	5	40	89.9	17.9	2.6 (2.9)	3.01±0.14; 238±44	4.0±0.4; (1.0±0.4)•10^5^
**10X**	6	42	90.2	19.7	2.0 (2.2)	3.90±0.15	6.3±0.5

**Fig 2:**
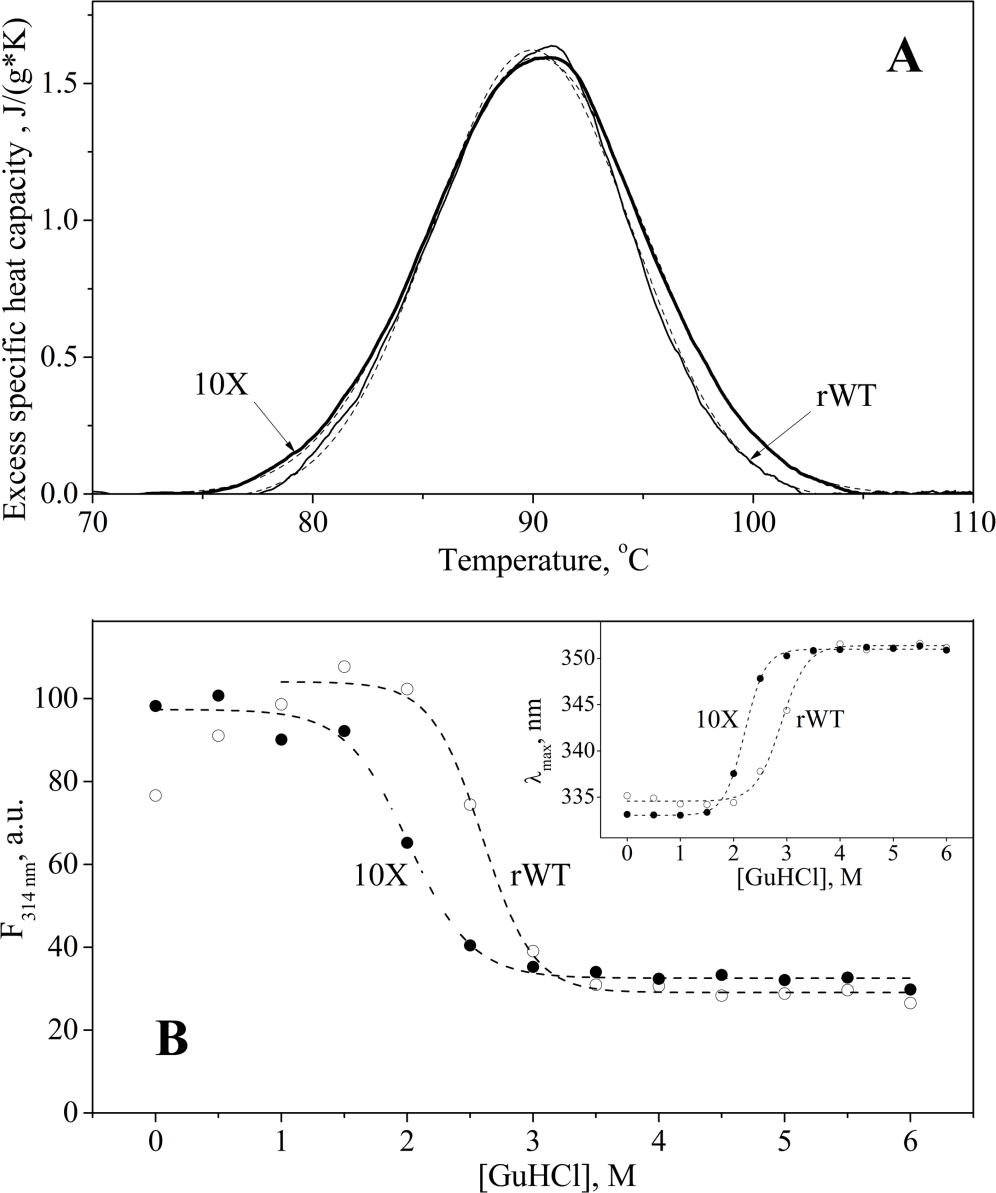
Stability of L35Ae samples towards thermal and chemical denaturation according to DSC (A) and intrinsic fluorescence (B) data, respectively. **(A)** The temperature dependencies of excess specific heat capacity for rWT L35Ae (solid curve) and L35Ae 10X (bold solid curve) were fitted by a single Gaussian function (dashed curves). Protein concentrations were 0.65 mg/ml and 1.5 mg/ml, for rWT L35Ae and L35Ae 10X samples, respectively. Buffer conditions: 20 mM H_3_BO_3_–NaOH, 300 mM NaCl, pH 8.5. Heating rate was 1 K/min. **(B)** GuHCl-induced unfolding of L35Ae samples followed by fluorescence intensity at 314 nm and position of tryptophan fluorescence spectrum maximum (inset). Excitation wavelength was 280 nm. Protein concentration was 3 μM. Buffer conditions: PBS, 150 mM NaCl, pH 7.0. The dashed curves are theoretical fits to the experimental data using Boltzmann function (Table 2).

**Fig 3:**
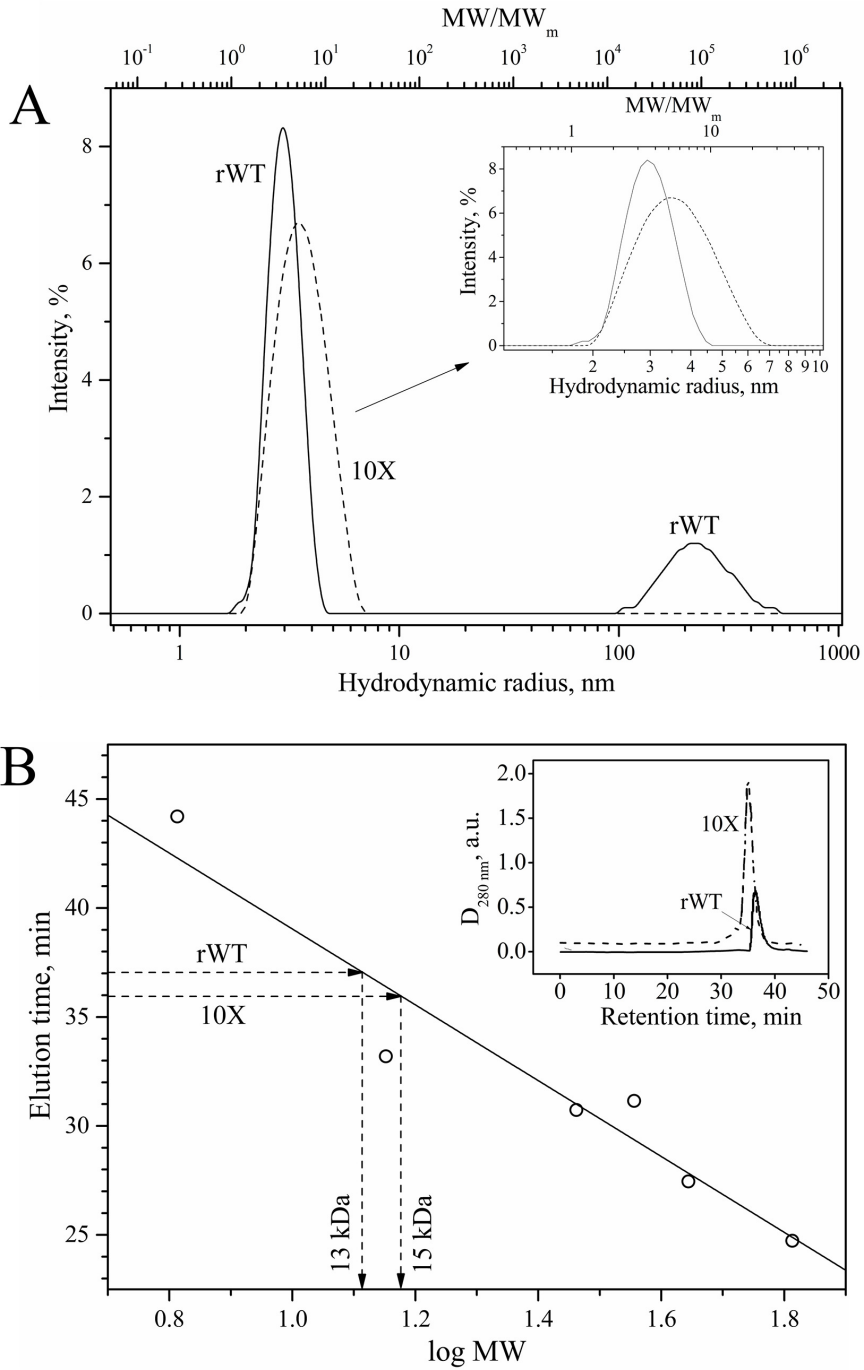
Hydrodynamic properties of rWT L35Ae and L35Ae 10X samples, according to DLS and high-resolution gel-filtration data. Buffer conditions: PBS, 150 mM NaCl, pH 7.0. **(A)** Hydrodynamic radius and extent of oligomerization measured for L35Ae samples (1.5 mg/ml) by DLS at 25.0°C. The extent of protein oligomerization is estimated as a ratio of apparent molecular weight of a protein (*MW*) and molecular weight of its monomer (*MW*_*m*_). The protein solutions were preliminarily passed through 10 nm filter. **(B)** Gel-filtration chromatography data for L35Ae samples (3.5–3.7 mg/ml) and molecular mass standards at room temperature (Superdex 75 10/300 GL column). The elution profiles of L35Ae samples are shown in the inset. The open circles corresponding to elution times of the mass standards were described by linear function (solid line). The apparent molecular masses of L35Ae samples were determined by comparison of their elution times with the calibration line, as indicated by dashed arrowed lines.

The contents of α-helices and β-sheets were estimated from far-UV CD data using CDPro software [55]. The parameters of thermal denaturation of the proteins were estimated from the DSC data presented in Fig 2A. Resistance of L35Ae to GuHCl (*[GuHCl]*_*1/2*_) was estimated from the spectrofluorimetric data shown in Fig 2B from fluorescence intensity or λ_max_ data. The *[GuHCl]*_*1/2*_ values calculated from λ_max_ data are indicated in parentheses. The mean hydrodynamic radius (*R*_*h*_) and mean extent of protein oligomerization (*MW/MW*_*m*_) were estimated from the DLS data, shown in Fig 3A. The mean extent of protein oligomerization is calculated as a ratio of mean apparent molecular weight of a protein (*MW*) and the molecular weight of its monomer (*MW*_*m*_, 10,988 Da for rWT L35Ae and 10,862 Da for L35Ae 10X). The CD experiments were carried out at 25°C in buffer: 5 mM Na_2_HPO_4_, 0.88 mM KH_2_PO_4_, 150 mM NaCl, 1.35 mM KCl, pH 7.0, at protein concentration of 11–12 μM. The experimental conditions for DSC, fluorimetric and DLS measurements are indicated in the captions to Fig 2 and Fig 3A.

The structural stability of rWT L35Ae was examined by differential scanning calorimetry (DSC) and a GuHCl-induced unfolding of the protein monitored by fluorescence emission of its single Trp residue. The temperature dependence of excess specific heat capacity for rWT L35Ae derived from DSC data (Fig 2A) showed a single heat sorption peak with a maximum at 90°C, which is characteristic for hyperthermophilic proteins [36]. Notably, the protein unfolding was irreversible (data not shown), which precluded rigorous equilibrium thermodynamic analysis of the process. Hence, the parameters of protein unfolding shown in Table 2 represent just apparent values. Despite high thermal stability of rWT L35Ae, its GuHCl-induced unfolding monitored by fluorescence intensity and position of tryptophan fluorescence spectrum maximum, λ_max_ (Fig 2B), demonstrated a moderate resistance of the protein to GuHCl with mid-transition at 2.6 M of GuHCl (Table 2).

rWT L35Ae propensity to multimerization/aggregation was studied using dynamic light scattering (DLS), chemical crosslinking with glutaraldehyde, and high-resolution gel-filtration. The protein sample passed through 10 nm filter exhibited at 25.0°C two DLS peaks (Fig 3A) corresponding to the major population with hydrodynamic radii (*R*_*h*_) ranging from 2 nm to 4.5 nm, and a minor population with *R*_*h*_ values from 100 nm to 500 nm. The major peak covers rWT L35Ae forms from monomer to octamer (Fig 3A) with mean extent of protein oligomerization of 4.0±0.4 (Table 2). Crosslinking of non-filtered sample of rWT L35Ae at 37°C with glutaraldehyde (Table 3) indicated that the protein mostly exists as a mixture of monomer, dimer, and a high molecular weight fraction not penetrating the resolving 4% gel. The prevalent contribution of the monomeric protein was evidenced by gel-filtration (Fig 3B).

**Table 3:** Distributions of oligomeric forms for rWT L35Ae and L35Ae 10X proteins measured by SDS-PAGE (4–15%) of the proteins, subjected to crosslinking with 0.05% glutaraldehyde at 37°C for 1 h.

L35Ae sample		Content of protein forms				
	Protein concentration, mg/ml	Monomer, %	Dimer, %	Trimer, %	Oligomers (30–100 kDa), %	High molecular weight fraction ^*^, %
**rWT**	1.0	46	15	6	1	33
**10X**	1.0	26	26	12	23	12
**rWT**	0.3	52	19	5	5	20
**10X**	0.3	36	22	10	24	8

Buffer conditions: PBS, 150 mM NaCl, pH 7.0.

^*^protein oligomers/aggregates not penetrating the resolving 4% gel

### Interaction of rWT L35Ae with HEK293 cells

**Fig 4:**
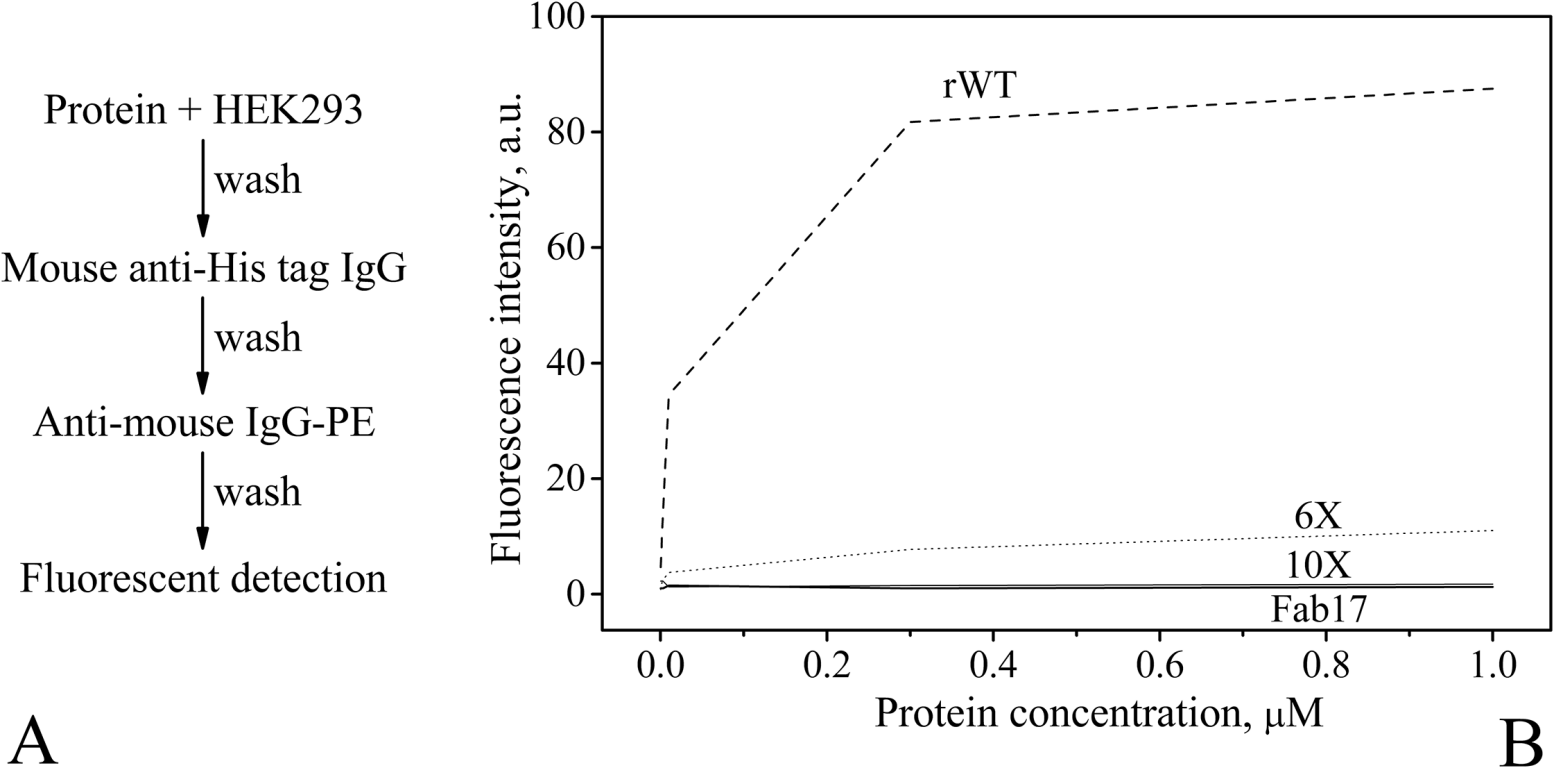
The scheme of preparation of HEK293 cells for flow cytometry experiment (A) and flow cytometry data for the HEK293 cells versus protein concentration (B). HEK293 cells were incubated with 6×His-tagged L35Ae or anti-hCCR7 Fab17 used as a negative control, followed by immunostaining. FACS buffer contained PBS, 150 mM NaCl, pH 7.0, 1–1.5% calf serum, and 0.01% NaN_3_. R-Phycoerythrin (PE) fluorescence was excited at 532 nm and detected at 580–583 nm.

The propensity of rWT L35Ae to multimerization and aggregation (Tables 2, 3 and Fig 3A) may favor non-specific interactions with biological structures (“stickiness”), thereby precluding its therapeutic and diagnostic alternative frame applications [48]. For this reason, rWT L35Ae was tested for non-specific binding to the surface of a model mammalian cell line, Human Embryonic Kidney 293 (HEK293). The HEK293 cells were incubated with 6×His-tagged rWT L35Ae or anti-hCCR7 Fab17 used as a negative control, followed by immunostaining and flow cytometry analysis (Fig 4). The incubation of 0.3–1 μM rWT L35Ae with HEK293 cells resulted in 50-55-fold higher fluorescence intensity of the immunolabeled protein, compared to Fab17, evidencing basic non-specific binding of rWT L35Ae to HEK293 cell surfaces.

### Mutagenesis of rWT L35Ae for suppression of its unspecific interaction with HEK293 cells

We carried out site-directed mutagenesis of rWT L35Ae to compensate for its excessive positive charge at neutral pH (+11.8 at pH 7.0, Table 4) and to decrease hydrophobic solvent accessible surface area (SASA) of the L35Ae molecule. The residues selected for substitution were chosen by visual inspection of model structures built on the basis of 2lp6 structure of L35Ae from *P*. *furiosus* (Fig 1B). To minimize a possible loss in the structural stability of L35Ae induced by the mutagenesis, energetically favorable mutations were chosen using the Rosetta.design server (http://rosettadesign.med.unc.edu/). Six substitutions of surface residues of rWT L35Ae were selected in this way: R12K, V22E, K47R, V53D, R60E, and R69K (Fig 1). As a result, a chain of alternating oppositely charged residues was formed, including residues K12, E22, K69, E60 (Fig 1B). The resulting mutant (referred to as ‘L35Ae 6X’) was predicted to exhibit a lowered fraction of hydrophobic SASA, a decreased charge at pH 7.0 (more neutral pI value), and efficiently compensated unfavorable charge-charge interactions (estimated using DelPhi module of the Discovery Studio Software, Accelrys Software Inc.), compared to rWT L35Ae (Table 4).

**Table 4:** Physico-chemical properties of the recombinant forms of L35Ae from *P*. *horikoshii* used in the present study.

L35Ae form	pI	Charge at pH 7.0	E_q-q_, kT	Fraction of hydrophobic SASA, %
**rWT**	10.6	+11.8	91	55.5
**6X**	9.9	+7.8	63	55.0
**10X**	9.3	+ 4.8	57	56.5

pI and charge at pH 7.0 were calculated using Protein Calculator v3.4 online service (http://protcalc.sourceforge.net/). The total energy of charge-charge interactions (E_q-q_) was calculated using DelPhi module of the Discovery Studio Software (Accelrys Software Inc.). The fraction of hydrophobic SASA was calculated using POPS online service [49]. The structural models of L35Ae forms were built based on PDB entry 2lp6 [50] for L35Ae from *P*. *furiosus* (Fig 1).

L35Ae 6X mutant protein was expressed in *E*. *coli*, isolated and purified to homogeneity. We then compared non-specific binding of L35Ae 6X and rWT L35Ae proteins to HEK293 cells using flow cytometry analysis. We found that at the concentrations up to 0.3–1 μM the L35Ae 6X bound to HEK293 cells up to 8-10-fold less efficiently compared to the rWT L35Ae protein (Fig 4). Thus, the compensation of excessive positive charge and hydrophobic SASA of L35Ae was found to be an effective way of reducing the protein’s propensity to non-specific interactions with complex biological surfaces, such as the surface of HEK293 cells.

The substitutions of surface residues of L35Ae 6X mutant designed to suppress its interaction with HEK293 cells were selected in a similar way: R3Y, I4V, R65T, and R71T (Fig 1). The resulting mutant, referred to as ‘L35Ae 10X’, was predicted to possess a decreased total charge at pH 7.0, and more optimal charge-charge interactions, compared to L35Ae 6X (Table 4). The sample of L35Ae 10X mutant was also expressed in *E*. *coli*, isolated and purified. Flow cytometry analysis run as above demonstrated that L35Ae 10X binds to HEK293 cells at negligible level, similar to that of the negative control, anti-hCCR7 Fab17 (Fig 4). Hence, the rational mutagenesis of L35Ae abolished its binding to HEK293 cells.

### Structural properties of L35Ae 10X mutant

To ensure that the mutagenesis of rWT L35Ae suppressing its interaction with HEK293 cells did not distort structure of the protein, the resulting L35Ae 10X mutant was studied by variety of physico-chemical techniques.

Far-UV CD measurements demonstrated that the secondary structure of L35Ae 10X mutant was equivalent to that of rWT L35Ae (Table 2). The temperature dependence of excess specific heat capacity of L35Ae 10X closely resembled the same of rWT L35Ae (Fig 2A): a single heat sorption peak with a maximum at 90°C. Similarly, protein unfolding was irreversible (data not shown). The GuHCl-induced unfolding of L35Ae 10X mutant, monitored by fluorescence intensity and λ_max_ value (Fig 2B), showed that it is less resistant to denaturation by GuHCl when compared to rWT L35Ae (*[GuHCl]*_*1/2*_ = 2.0 M versus 2.6 M for rWT L35Ae, Table 2). The lowered resistance to GuHCl may arise from optimized charge-charge interactions within the L35Ae 10X molecule (Table 4), which makes the protein molecule more susceptible to the destabilizing action of charged GuHCl molecules.

A comparative DLS study of rWT and 10X mutant L35Ae samples filtered through 10 nm filter showed that L35Ae 10X protein lacked the high molecular weight fraction that was observed with the rWT L35Ae protein (Fig 3A). The DLS spectrum of L35Ae 10X exhibited a broad single peak with hydrodynamic radii ranging from 2 nm to 7 nm. This suggests that L35Ae 10X exists in solution as a mixture of forms spanning from the monomer to about a 20-mer (Fig 3A, inset). The mean extent of the protein oligomerization was 6.3±0.5, which markedly exceeded that for rWT L35Ae (Table 2). Thus, the L35Ae mutagenesis suppressed aggregation, but promoted the oligomerization of the protein. This conclusion was further confirmed by crosslinking of the non-filtered sample of the L35Ae 10X protein with glutaraldehyde (Table 3). Compared to rWT L35Ae, L35Ae 10X mutant exhibited lower contents of the monomer and the oligomers/aggregates not penetrating the resolving SDS-PAGE gel, but higher contents of the trimer and the oligomers with molecular mass of 30–100 kDa. The increased propensity of L35Ae 10X to oligomerization is further confirmed by high-resolution gel-filtration (Fig 3B). The apparent molecular mass of L35Ae 10X sample estimated by gel-filtration exceeds that for rWT L35Ae protein (about 15 kDa and 13 kDa for the mutant and wild type proteins, respectively). Overall, the L35Ae 10X mutant was less susceptible to aggregation, but was more prone to the oligomerization. The latter feature of L35Ae 10X could be due to its lowered molecular charge (Table 4) and respectively compensated electrostatic repulsion of the protein molecules.

## Discussion

Remarkably high structural stability of proteins originating from extremophilic organisms [34–36] makes them attractive candidates for alternative scaffold engineering, as they should tolerate massive structural alterations [32, 33]. Indeed, some of the developed APSs are based on proteins originating from extremophiles (Sso7d protein from *S*. *solfataricus* [37, 38] and Sac7d from *S*. *acidocaldarius* [39, 40]). Here, the 50S ribosomal protein L35Ae from hyperthermophilic archaea *P*. *horikoshii* [41] was explored with regard to its potential for creation of artificial binding proteins, including structural stability [32], propensity to multimerization and aggregation [48], and association with model mammalian cells. The choice of L35Ae protein is advantageous due to its unique fold, containing three CDR-like loops (Fig 1) suited for randomization, and ease of bacterial production.

Thermal unfolding of rWT L35Ae was accompanied by a cooperative heat of sorption (Fig 2A) with mid-transition temperature, *T*_*0*_, of 90°C (Table 2), which is equivalent to that for Sac7d protein (Table 5) and markedly exceeds those for wild type samples of such well-established APSs as Anticalin, Affibody and Adnectin (61–84°C) [37]. Meanwhile, in contrast to some other proteins originating from *P*. *horikoshii* (acylphosphatase [61], aromatic aminotransferase [62] and CutA1 protein [63]) *T*_*0*_ value for rWT L35Ae was below the optimal growth temperature of *P*. *horikoshii*, 98°C [41], which suggests an additional stabilization of the protein structure under *in vivo* conditions, likely due to its interaction with tRNA and/or ribosomal proteins.

**Table 5:** Comparison of structural features of L35Ae 10X, Sso7d and Sac7d proteins (see Table 2).

Protein	Number of residues	T_0_, °C	[GuHCl]_1/2_, M	Propensity to oligomerization	References
**L35Ae 10X**	88	90.2	2.0	yes	Present study
**Sso7d**	60	98	4	no	[37, 38]
**Sac7d**	66	90.7	2.8	no	[64, 65]

Despite its extremely high thermal stability, rWT L35Ae demonstrates a moderate resistance to guanidine hydrochloride with *[GuHCl]*_*1/2*_ of 2.6 M (Fig 2B, Table 2), which is equivalent to that for extremophilic Sac7d protein (Table 5). Similarly disproportionate protein resistance to GuHCl and thermal stability were reported for other proteins originating from extremophiles, including elongation factor 1α from thermoacidophilic archaea *Sulfolobus solfataricus* (*T*_*0*_ of 95°C**,** *[GuHCl]*_*1/2*_ **=** 2.7–2.9M [66, 67]) and two thermophilic esterases (*T*_*0*_ = 91–99°C**,** *[GuHCl]*_*1/2*_ = 1.9–2.1 M [68]). Since ion pairs are considered to be a dominant factor stabilizing hyperthermophilic proteins [35, 69, 70], the charged GuHCl molecules are expected to disrupt the stabilizing charge-charge interactions, resulting in relatively low resistance of hyperthermophilic proteins towards GuHCl [68]. Nonetheless, the structural stability of rWT L35Ae is analogous to that for Sac7d protein.

Though L35Ae protein from *P*. *furiosus* was shown to be monomeric in solution [50], rWT L35Ae is susceptible to oligomerization and aggregation at the protein concentrations of 0.3–1.5 mg/ml, as confirmed by chemical crosslinking (Table 3) and DLS (Table 2, Fig 3A). The propensity to oligomerization was reported for other ribosomal proteins, including P1 and P2 proteins [71, 72]. Furthermore, hyperthermophilic proteins tend to have a higher oligomerization state than their mesophilic homologues, which seems to favor their structural stability [34, 63, 73]. Meanwhile, thermophilic Sso7d and Sac7d proteins were shown to be monomeric in solution (Table 5). Although a vast majority of alternative protein scaffolds possesses monomeric structure, examples of oligomeric APSs such as Avimer and Tetranectin [2] indicate that propensity of rWT L35Ae to oligomerization may also be advantageous for the development of an APS, when taking into account the avidity effect [74]. Meanwhile, the non-specific nature of L35Ae oligomerization may negatively affect the avidity effect. Potential involvement of the region of CDR-like loops of L35Ae protein in the oligomerization could prevent target recognition. Nevertheless, diversification of the CDR-like loops in this case may inhibit oligomerization of the protein.

Application of artificial binding proteins for therapeutic/diagnostic purposes dictates lack of their non-specific binding to complex biologically relevant surfaces, including surfaces of human cells. Meanwhile, rWT L35Ae protein demonstrated a marked association with surface of HEK293 cells, as confirmed by the flow cytometry analysis (Fig 4). This negative feature of rWT L35Ae could be related to its intrinsic propensity to aggregation (Table 3) and to excessive positive charge of the protein molecule (Table 4). The rational design of rWT L35Ae aimed at compensation of its positive charge and decreasing its hydrophobic SASA indeed resulted in the L35Ae 6X mutant (Fig 1) with drastically reduced non-specific binding to HEK293 cells (Fig 4B). Further decreased positive charge in the L35Ae 10X mutant molecule completely abolished the protein binding to HEK293 cells (Fig 4B), despite its higher hydrophobic SASA (Table 4). This indicates that the uncompensated positive charge of rWT L35Ae molecule, which potentially promotes its interaction with tRNAs [43], is also crucially linked to non-specific binding of L35Ae to HEK293 cells.

Though increased hydrophobic SASA of L35Ae 10X mutant (Table 4) could favor its self-association and aggregation, chemical crosslinking data evidence that the mutant is less susceptible to aggregation compared to rWT L35Ae (Table 3). Nevertheless, L35Ae 10X mutant forms higher order oligomers, as confirmed by DLS (Table 2, Fig 3A) and gel-filtration (Fig 3B). The latter effect can be explained by the 2.5-fold lower molecular charge of the L35Ae 10X mutant (Table 4), which may decrease the electrostatic component of the energetic penalty of its oligomerization. The lowered content of the aggregated forms of 10X mutant could be due to specific structure of the protein oligomers, which prevents formation of the higher order structures leading to accumulation of the aggregates.

The extensive mutagenesis of rWT L35Ae altered its propensity to self-association, but did not affect its secondary structure and thermal stability (Table 2, Fig 2A). Meanwhile, L35Ae 10X exhibits markedly lowered resistance to GuHCl (*[GuHCl]*_*1/2*_ is lowered by 0.6 M with regard to rWT L35Ae—Table 2, Fig 2B), which likely reflects optimized charge-charge interactions in L35Ae 10X mutant (Table 4) resulting in facilitated disruption of the protein structure by charged GuHCl molecules (see ref. [68]). The value of *[GuHCl]*_*1/2*_ for L35Ae 10X mutant is lower compared to that for Sso7d and Sac7d proteins (Table 5) and some other established APSs (10^th^ human fibronectin type III domain [75], DARPin [76], single-chain variable fragment [77], Top7 [78], *etc*.).

## Conclusions

The crucial features of a unique fold of 50S ribosomal protein L35Ae that possesses CDR-like loops attractive for randomization (Fig 1) were probed for the development of an alternative scaffold. Since structural stability is vital for APS design, L35Ae from hyperthermophilic archaea *P*. *horikoshii* was chosen for further investigation. Indeed, rWT L35Ae is extremely thermally stable, however it exhibits a marked propensity to oligomerize/aggregate and interact with HEK293 cells. The rational mutagenesis of rWT L35Ae aimed at suppression of its excessive positive molecular charge gave rise to L35Ae 10X mutant with native-like secondary structure and thermal stability, a lowered susceptibility to aggregation and abolished HEK293 cell binding. The increased propensity of L35Ae 10X to oligomerization could prevent target recognition, but may also provide an advantage with respect to monomeric APSs due to the avidity effect. The resistance of L35Ae 10X mutant to GuHCl is lowered compared to established APSs. In summary, L35Ae 10X mutant’s properties show thermal stability resembling or exceeding that for established alternative protein scaffolds, lowered resistance to GuHCl and well-defined propensity to multimerization, which may prevent its use as an APS. This example of L35Ae shows that despite their outstanding thermal stability, proteins originating from extremophilic organisms may possess some problematic features, complicating their direct use in biotechnological applications, including relatively low stability to charged chemical denaturants and noticeable tendency to multimerization.
